# PDE3B and HBB are key prognostic biomarkers driving cell proliferation and regulating immune microenvironment in breast cancer

**DOI:** 10.1186/s41065-025-00470-z

**Published:** 2025-06-03

**Authors:** Bolong Yin, Xiangrong Luo, Xuebo Yan, Hui Shen, Jianping Jiang

**Affiliations:** The Central Hospital of Shaoyang, Shaoyang Hunan, 422400 China

**Keywords:** Breast cancer, Single-cell RNA-seq, TCGA, Prognostic biomarkers, PDE3B, HBB, Tumor microenvironment, Immune modulation, LASSO regression, Cell proliferation

## Abstract

**Background:**

Breast cancer is a heterogeneous malignancy with diverse tumor subpopulations and complex tumor-immune interactions. This study explores the prognostic and functional roles of PDE3B and HBB in breast cancer, focusing on their contributions to proliferation and immune microenvironment modulation.

**Methods:**

Single-cell RNA sequencing (scRNA-seq) and TCGA data were analyzed to identify malignant subpopulations and prognostic genes. Differential gene expression, KEGG enrichment, LASSO regression, and Kaplan-Meier survival analyses were performed. Immune infiltration was assessed using EPIC deconvolution. Functional validation included qRT-PCR, IHC, Western blot, and proliferation assays in MDA-MB-231 cells.

**Results:**

Malignant cell type 3 exhibited the highest proliferative potential. PDE3B and HBB were identified as prognostic markers, strongly associated with poor survival and immune cell infiltration. Overexpression of these genes enhanced proliferation, while their knockout suppressed it.

**Conclusion:**

PDE3B and HBB drive breast cancer proliferation and immune modulation, making them promising biomarkers and therapeutic targets. Further research should assess their potential in targeted therapies.

**Supplementary Information:**

The online version contains supplementary material available at 10.1186/s41065-025-00470-z.

## Introduction

Breast cancer is one of the most prevalent malignancies worldwide, characterized by significant heterogeneity in its molecular and cellular landscape [1]. Despite advances in early detection and therapeutic interventions, the mechanisms driving breast cancer progression and treatment resistance remain incompletely understood. Identifying key molecular drivers and understanding their roles in tumor heterogeneity and proliferation are critical for improving prognosis and developing targeted therapies.

The Cancer Genome Atlas (TCGA) has provided a wealth of genomic, transcriptomic, and epigenomic data from thousands of cancer patients, enabling researchers to identify key molecular alterations and their clinical implications across various cancer types, including breast cancer. Numerous studies utilizing TCGA data have highlighted the roles of specific genes, pathways, and immune components in breast cancer progression. For example, TCGA-based analyses have been instrumental in identifying PAM50 molecular subtypes, elucidating the impact of tumor-infiltrating immune cells, and uncovering potential prognostic markers [2]. While these studies have provided significant insights, there remains a need to link these large-scale findings to functional mechanisms driving tumor heterogeneity and proliferation at the single-cell level.

Single-cell RNA sequencing (scRNA-seq) has emerged as a powerful tool for dissecting cellular heterogeneity in tumors [3, 4]. By analyzing individual cells, this technology provides insights into distinct subpopulations and their functional states within the tumor microenvironment. Proliferative potential, a hallmark of cancer, varies among cell subpopulations and is closely linked to tumor aggressiveness. Investigating the pathways underlying these differences can help uncover novel therapeutic targets. In addition to tumor cell-intrinsic factors, interactions between cancer cells and the immune microenvironment significantly influence tumor progression. Immune cell infiltration and the expression of immune-related genes often correlate with prognosis and response to therapy [5]. Integrating genomic data from large cohorts, such as TCGA, with single-cell analyses can reveal key genes driving tumor-immune interactions and their clinical relevance.

This study focuses on identifying and validating two genes, PDE3B and HBB, that were implicated in breast cancer progression through an integrative analysis of scRNA-seq, TCGA, and an independent patient cohort. PDE3B and HBB were found to play critical roles in tumor proliferation and immune microenvironment modulation [6, 7]. Functional validation in MDA-MB-231 cell lines, including overexpression and knockout experiments, demonstrated their contributions to promoting cell proliferation. By combining bioinformatics analysis and experimental validation, this work highlights PDE3B and HBB as potential prognostic biomarkers and therapeutic targets for breast cancer, providing a deeper understanding of the molecular mechanisms underlying tumor heterogeneity and progression.

## Methods

### Single-cell RNA sequencing analysis

Single-cell RNA sequencing (scRNA-seq) data were E-MTAB-8107, previously published in EMBL-EBI. The scRNA-seq data from E-MTAB-8107 were analyzed using the Seurat R package (version 4.0). Quality control was performed to filter out low-quality cells with fewer than 200 or more than 5,000 detected genes and cells with a mitochondrial gene fraction greater than 10%. The data were normalized, and variable features were identified for dimensionality reduction using principal component analysis (PCA). Unsupervised clustering was performed using the Louvain algorithm, and the results were visualized using Uniform Manifold Approximation and Projection (UMAP). Malignant cell types were annotated based on the expression of well-established marker genes frequently used in breast cancer studies. These included EPCAM (epithelial cell adhesion molecule), a marker highly expressed in malignant epithelial cells, and KRT8/18 (keratins 8 and 18), which are luminal epithelial markers commonly elevated in breast cancer. MKI67, a well-known marker for cell proliferation, was utilized to distinguish actively dividing tumor cells, while TP63, a basal epithelial marker, was particularly relevant for identifying basal-like subtypes, such as triple-negative breast cancer. To identify distinct subpopulations of malignant cells, unsupervised clustering was performed using the Louvain algorithm implemented in the Seurat R package. Dimensionality reduction was achieved with principal component analysis (PCA), and clusters were visualized using Uniform Manifold Approximation and Projection (UMAP). These methods allowed for the identification of unique cellular subpopulations based on transcriptional profiles. Proliferative capacity was assessed using a previously published gene set associated with cell cycle and proliferation processes. Key genes in this set included MKI67, a direct marker of cell proliferation, and PCNA (proliferating cell nuclear antigen), which is crucial for DNA replication. Additionally, CCNA2 and CCNB1 (cyclins A2 and B1) were included as they play vital roles in cell cycle regulation. CDK1 (cyclin-dependent kinase 1), essential for the G2/M phase transition, was also incorporated, along with TOP2A (topoisomerase II alpha), which is critical for DNA replication and chromosome segregation, and AURKA (aurora kinase A), a mitotic regulator frequently upregulated in cancers. Proliferation scores were calculated by integrating the expression levels of these genes, normalized across all cells. Higher proliferation scores identified subpopulations with enhanced proliferative potential, providing insights into the biological heterogeneity of malignant cells.

### Differential gene expression analysis

Differential expression analysis was performed on TCGA breast cancer data obtained from the UCSC Xena database. Tumor and adjacent normal tissue samples were compared using the DESeq2 R package. Genes with a|log2 fold change| > 1 and adjusted p-value < 0.05 were considered differentially expressed. Volcano plots were generated to highlight significantly upregulated and downregulated genes. Intersection analysis was conducted to identify genes shared between TCGA tumors and malignant cell type-specific markers identified from scRNA-seq data.

### KEGG pathway enrichment analysis

Key genes identified from scRNA-seq clustering and differential expression analyses were subjected to Kyoto Encyclopedia of Genes and Genomes (KEGG) pathway enrichment analysis using the clusterProfiler R package. Pathways with an adjusted p-value < 0.05 were considered significantly enriched. Results were visualized as dot plots, with dot size representing the number of genes involved in each pathway and color indicating significance.

### LASSO regression and survival analysis

LASSO (Least Absolute Shrinkage and Selection Operator) regression was performed using the glmnet R package to identify prognostic genes among the intersecting gene set. The optimal λ value was determined using 10-fold cross-validation. Kaplan-Meier survival analysis was conducted on breast cancer patients, stratifying them into high- and low-expression groups based on the median expression levels of the identified genes. Survival curves were generated using the survminer R package, and the log-rank test was used to assess statistical significance. Time-dependent ROC curves were generated to evaluate the prognostic accuracy of the identified genes using the timeROC R package.

### Immune cell infiltration analysis

Immune cell infiltration in TCGA breast cancer samples was assessed using EPIC (Estimating the Proportion of Immune and Cancer Cells) deconvolution. Correlation analysis between gene expression levels and immune cell fractions was performed using Pearson’s correlation. Results were visualized as scatter plots and heatmaps, with significant correlations (*p* < 0.05) marked.

### Independent cohort validation

An independent patient cohort of breast cancer tissues was collected for validation. Gene expression levels were determined using quantitative real-time PCR (qRT-PCR). Clinicopathological characteristics were recorded, and Kaplan-Meier survival analysis was performed to assess the prognostic relevance of the genes. Time-dependent ROC curves were generated to evaluate 5-year survival prediction accuracy.

### Independent cohort patient recruitment and inclusion criteria

An independent cohort of breast cancer patients was recruited from The Central Hospital of Shaoyang between 2016 and 2022. Inclusion criteria were: (1) histologically confirmed breast cancer; (2) no prior neoadjuvant therapy; (3) available clinical and pathological data; and (4) sufficient tissue samples for analysis. Patients with other malignancies, severe comorbidities, or incomplete clinical data were excluded. A total of 342 patients were included in the study. The study was approved by The Central Hospital of Shaoyang Ethics Committee (Approval No. 2022033). Written informed consent was obtained from all participants prior to sample collection and data analysis, in accordance with the Declaration of Helsinki. Patient confidentiality and data security were maintained throughout.

### Sample collection and processing

Fresh tumor tissue samples were obtained during surgical procedures. Samples were divided into two parts: one part was stored in liquid nitrogen for RNA extraction, and the other part was fixed in formalin and embedded in paraffin for immunohistochemistry (IHC) analysis. Clinicopathological characteristics, including tumor stage, lymph node involvement, molecular subtype, and metastasis status, were recorded for each patient. This independent cohort was used to validate the prognostic relevance of PDE3B and HBB, and their association with clinical outcomes such as overall survival and immune microenvironment modulation.

### Immunohistochemistry (IHC)

IHC was performed on paraffin-embedded tissue sections from the independent cohort. Sections were deparaffinized, rehydrated, and subjected to antigen retrieval. Primary antibodies against PDE3B and HBB were applied, followed by secondary antibody incubation and visualization with DAB substrate. Staining intensity and localization were evaluated by pathologists blinded to the study.

### Cell culture and genetic manipulation

The MDA-MB-231 breast cancer cell line was obtained from the American Type Culture Collection (ATCC) and maintained in DMEM supplemented with 10% fetal bovine serum (FBS) and 1% penicillin–streptomycin. Cells were cultured at 37 °C in a humidified atmosphere containing 5% CO₂.

For HBB and PDE3B overexpression, full-length coding sequences were cloned into the mammalian expression vector pcDNA3.1(+). Gene-specific primers were used to amplify each insert by PCR; products were purified, digested with EcoRI and XhoI, and ligated into pcDNA3.1(+). All constructs were confirmed by Sanger sequencing.

Knockdown of HBB and PDE3B was achieved using lentiviral shRNA vectors. Target sequences were cloned into pLKO.1-puro:

- HBB shRNA: 5′-GGACTTCCAGCAACCTCAA-3′
- PDE3B shRNA: 5′-GCAGCTGTACTACATCTTA-3′

Lentiviral particles were produced by co-transfecting HEK293T cells with the shRNA plasmid, psPAX2 packaging plasmid, and pMD2.G envelope plasmid using Lipofectamine 3000 (Thermo Fisher Scientific). Viral supernatants were harvested, filtered, and used to transduce MDA-MB-231 cells, which were then selected with 2 µg/mL puromycin for 7 days. Transfection and transduction efficiencies were confirmed by Western blotting and qRT-PCR.

### Quantitative Real-Time PCR (qRT-PCR)

Total RNA was extracted from cells using the RNeasy Mini Kit (Qiagen), and cDNA was synthesized using the High-Capacity cDNA Reverse Transcription Kit (Thermo Fisher Scientific). Gene expression levels were assessed using SYBR Green Master Mix (Applied Biosystems) on an ABI 7500 Real-Time PCR System. Primers used included:

- HBB Forward: 5’-CACATGTTGCCACACTGAGT-3’
- HBB Reverse: 5’-CACCAACTTCATCCACGTTC-3’
- PDE3B Forward: 5’-GGAGTCTGATCGTTTGCTTG-3’
- PDE3B Reverse: 5’-CCTTGCAGTATAGCCTCGTT-3’
- β-actin Forward: 5’-GCTCCTCCTGAGCGCAAG-3’
- β-actin Reverse: 5’-GCCCAATACGACCAAATCC-3’

Relative expression levels were calculated using the 2-ΔΔCt method, normalized to β-actin.

### Western blot analysis

Cells were lysed in RIPA buffer containing protease inhibitors (Roche). Protein concentrations were determined using a BCA Protein Assay Kit (Thermo Fisher Scientific). Equal amounts of protein (20 µg) were resolved on 12% SDS–PAGE gels and transferred to PVDF membranes. Primary antibodies were: anti-HBB (1:1000, Abcam), anti-PDE3B (1:1000, Abcam), and anti-GAPDH (1:5000, Sigma-Aldrich). Membranes were then incubated with HRP-conjugated secondary antibodies, and signals were detected using ECL reagent (Bio-Rad).

### CCK-8 proliferation assay

Cell proliferation was assessed using the Cell Counting Kit-8 (CCK-8) assay. Transduced MDA-MB-231 cells were seeded into 96-well plates at a density of 2,000 cells per well. At specified time points, CCK-8 reagent was added, and absorbance at 450 nm was measured using a microplate reader. Proliferation rates were calculated as fold changes in absorbance relative to baseline.

### Cell counting for proliferation analysis

For manual cell counting, MDA-MB-231 cells were seeded into 6-well plates at a density of 50,000 cells per well. Cells were harvested and counted using a hemocytometer at 0, 24, 48, and 72 h post-seeding. Counts were performed in triplicate for each condition—PDE3B and HBB overexpression, knockout, and control groups.

### Statistical analysis

All experiments were performed in biological triplicate, and data are presented as mean ± standard deviation (SD). Statistical analyses were conducted using GraphPad Prism (version 9.0). Differences between groups were assessed using Student’s t-tests or one-way ANOVA with post hoc Tukey’s test, as appropriate. Kaplan-Meier survival curves were compared using the log-rank test. Correlations between gene expression and immune cell fractions were evaluated using Pearson’s correlation coefficient. For all analyses, a p-value < 0.05 was considered statistically significant. For enrichment analysis, Benjamini–Hochberg multiple comparisons were used to adjust the p-value.

## Results

### Single-cell RNA-seq analysis reveals heterogeneity in malignant cell types and their proliferative potential

**Fig. 1:**
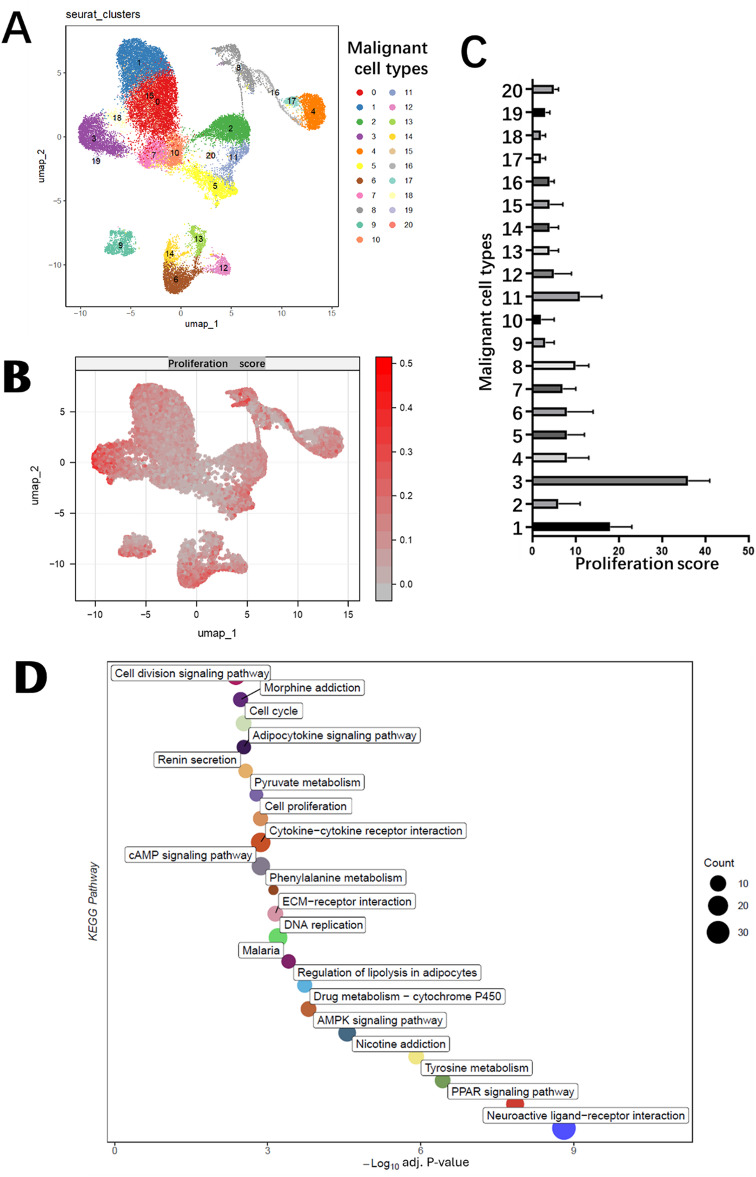
Single-cell analysis reveals heterogeneity in malignant cell proliferation and associated pathways. (**A**) UMAP visualization of Seurat clusters and malignant cell types. UMAP visualization of Seurat clusters. Each dot represents a single cell, colored by cluster. Malignant cell types are annotated and indicated by distinct shapes. (**B**) UMAP visualization of proliferation scores. Single cells were scored for proliferation potential using a gene set. The color gradient represents the proliferation score, with higher scores in red. (**C**) Comparison of proliferation scores across malignant cell types. The x-axis shows proliferation scores, and the y-axis lists malignant cell types. Error bars represent the standard error of the mean (SEM). (**D**) KEGG pathway enrichment analysis of malignant cell type 3. Dot plot shows -log10(adJ.p-value) for enriched pathways with Benjamini–Hochberg adjusted. Dot size represents the number of feature genes involved in each pathway

We performed unsupervised clustering of single cells using Seurat and visualized the results with UMAP, identifying 20 distinct cell clusters (Fig. 1A). Each cluster likely represents a unique cell subpopulation. Integrating malignant cell type annotations revealed that these malignant cells were distributed across multiple clusters, highlighting molecular heterogeneity among malignant cell types.

To evaluate proliferative capacity, we calculated proliferation scores (genes used for this calculation were listed in supplementary material Table 1) for each cell based on a previously published gene set. The proliferation scores varied significantly across clusters, indicating differences in proliferative potential among subpopulations (Fig. 1B). An analysis of proliferation scores across malignant cell types revealed distinct patterns. Notably, malignant cell type 3 exhibited significantly higher proliferation scores compared to other types, suggesting its enhanced proliferative capacity (Fig. 1C).

To explore the biological processes underlying this heterogeneity, we performed KEGG pathway enrichment analysis using feature genes of malignant cell type 3. The analysis revealed significant enrichment in pathways related to the cell cycle, DNA replication, and other proliferation-associated processes (Fig. 1D). These findings suggest that the observed heterogeneity in proliferation is driven by distinct molecular pathways in malignant cell subpopulations.

### Differential expression analysis reveals shared gene signatures between TCGA tumors and three malignant cell types

**Fig. 2:**
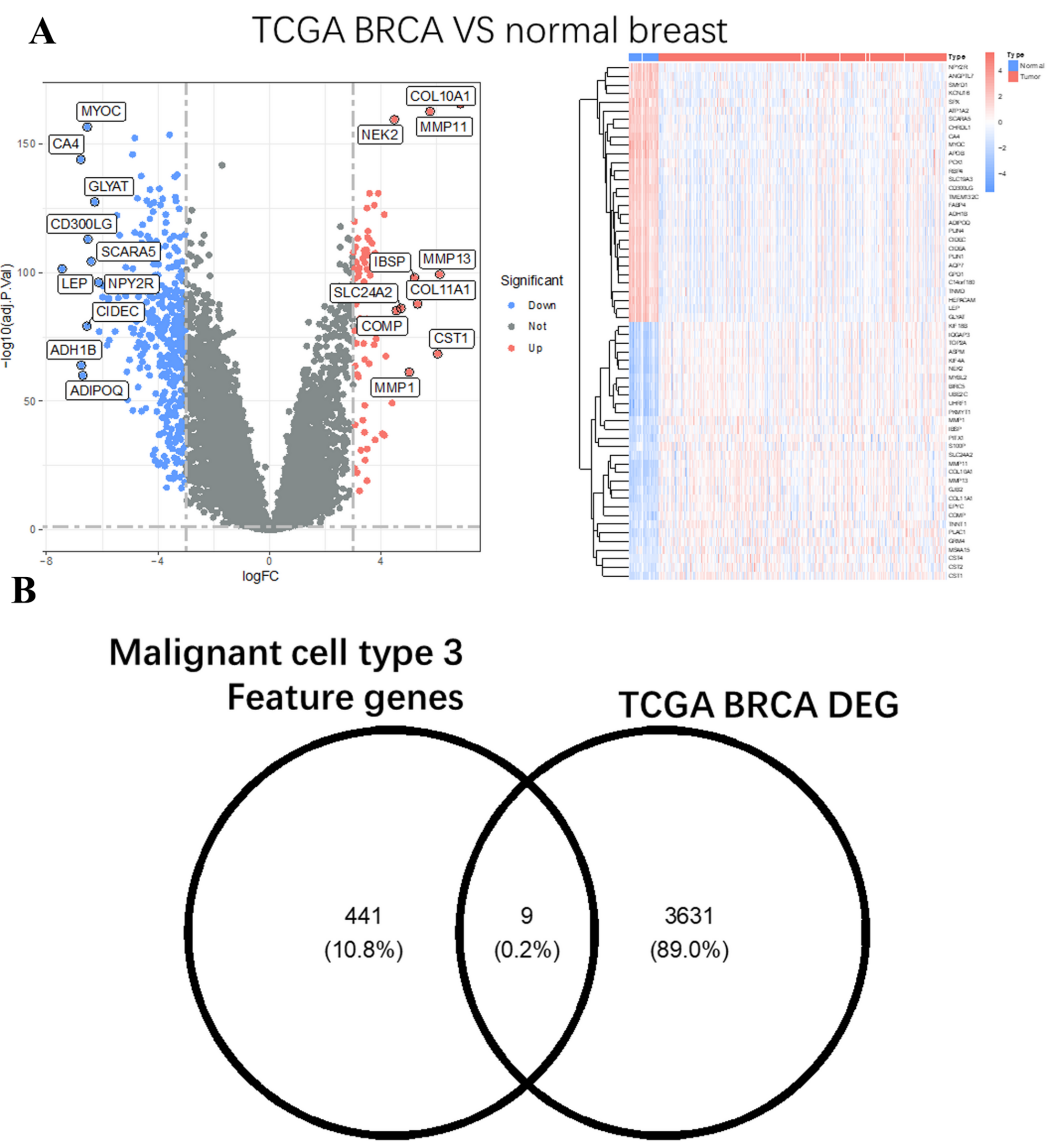
Differential gene expression analysis of TCGA tumors and their overlap with three malignant cell types. (**A**) Volcano plot of differentially expressed genes in TCGA tumors versus adjacent normal tissues. The x-axis represents the log2 fold change (logFC), and the y-axis represents -log10(p-value). Significantly upregulated genes are shown in red, downregulated genes in blue, and nonsignificant genes in gray. Annotated genes highlight those shared with malignant cell type-specific markers. (**B**) Heatmap of shared differentially expressed genes between TCGA tumors and malignant cell types. Each row represents a gene, and each column represents a tumor sample. Red indicates high expression, and blue indicates low expression, with hierarchical clustering used to group genes by expression pattern

To identify the shared molecular features between TCGA breast cancer tumors and the three identified malignant cell types, we performed a differential gene expression analysis. Volcano plots highlight significantly upregulated (red) and downregulated (blue) genes in TCGA tumors compared to adjacent normal tissues, with selected genes of interest annotated (Fig. 2A). Notably, several upregulated genes, including PDE3B and HBB, overlapped with malignant cell type-specific markers, suggesting these genes may play roles in tumor progression and proliferation.

Additionally, we visualized the expression of intersecting genes across tumor samples using a heatmap (Fig. 2B). The heatmap reveals distinct expression patterns, with genes grouped by their regulation status (upregulated or downregulated). Upregulated genes demonstrate consistent overexpression in tumors, supporting their relevance in malignant cell behavior.

These results highlight a subset of genes shared between TCGA tumors and the malignant cell types, providing insights into their potential roles in driving tumor heterogeneity and progression.

### Identification of prognostic genes using LASSO regression and validation with survival analysis in TCGA data

**Fig. 3:**
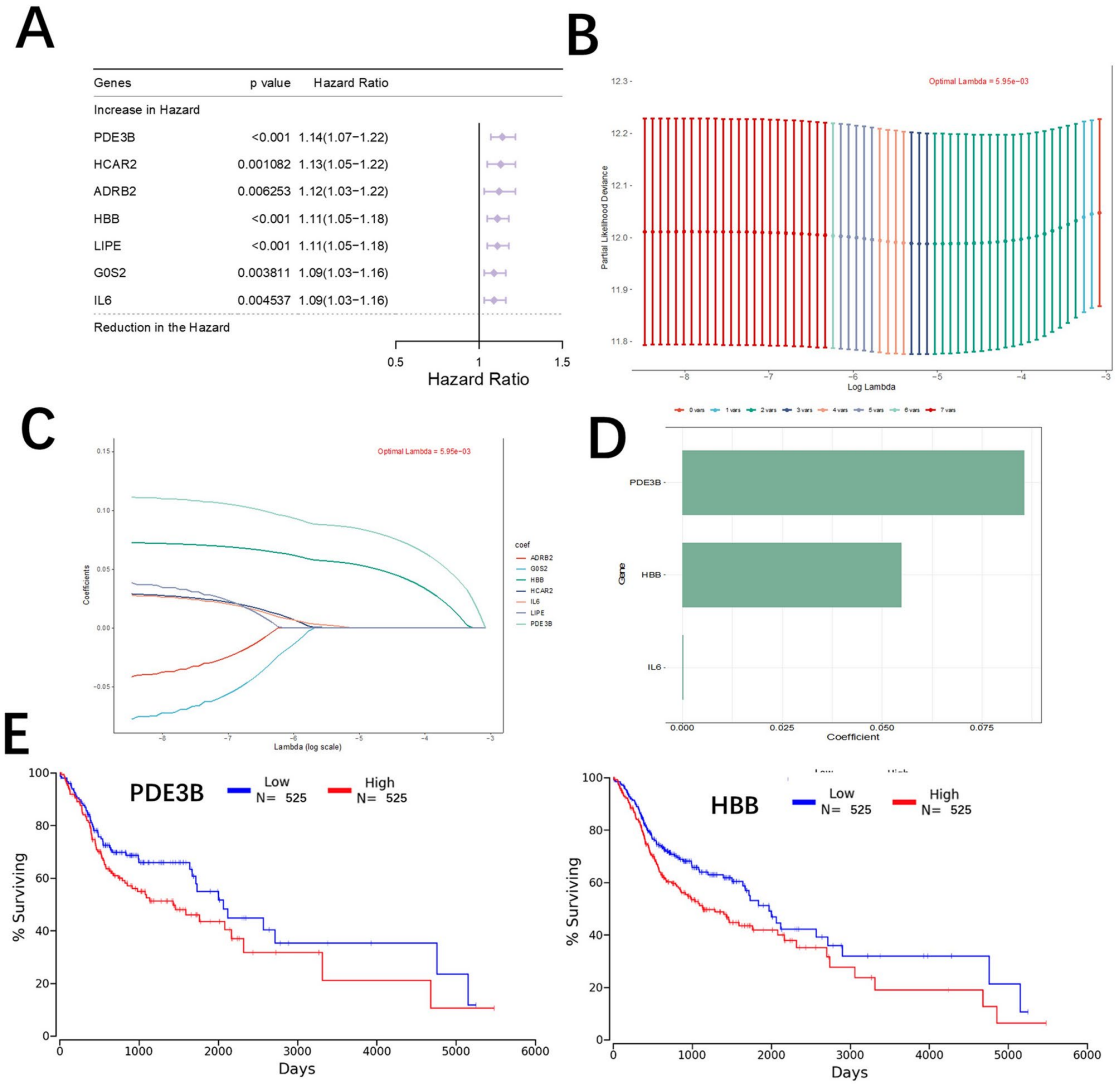
LASSO regression and survival analysis for prognostic gene identification. (**A**) LASSO coefficient profiles of intersecting genes. Each line represents a gene, and the x-axis shows the log(λ) value. (**B**) Tuning parameter (λ) selection in the LASSO model using 10-fold cross-validation. The red dot indicates the optimal λ value that minimizes the mean cross-validated error. (**C**) Bar plot of selected genes with nonzero coefficients in the LASSO model. PDE3B and HBB were identified as significant prognostic genes. (**D**) Kaplan-Meier survival analysis of PDE3B. Patients were stratified into high- and low-expression groups, and overall survival was compared using the log-rank test. (**E**) Kaplan-Meier survival analysis of HBB. Patients were stratified into high- and low-expression groups, and overall survival was compared using the log-rank test

To identify key prognostic genes among the intersecting gene set, we applied LASSO regression on TCGA breast cancer data. The optimal λ value was determined using 10-fold cross-validation, as shown in the tuning plots (Fig. 3A, B). This analysis identified two genes, PDE3B and HBB, with nonzero coefficients, indicating their potential roles in breast cancer prognosis (Fig. 3C). We validated the prognostic significance of these two genes using Kaplan-Meier survival analysis. Patients were stratified into high- and low-expression groups based on the median expression levels of PDE3B and HBB. Kaplan-Meier plots revealed that high expression of PDE3B and HBB was significantly associated with worse overall survival in TCGA patients (*p* < 0.05) (Fig. 3D, E). These findings suggest that PDE3B and HBB are potential prognostic markers for breast cancer.

### Association of PDE3B and HBB with immune cell infiltration in the tumor microenvironment

**Fig. 4:**
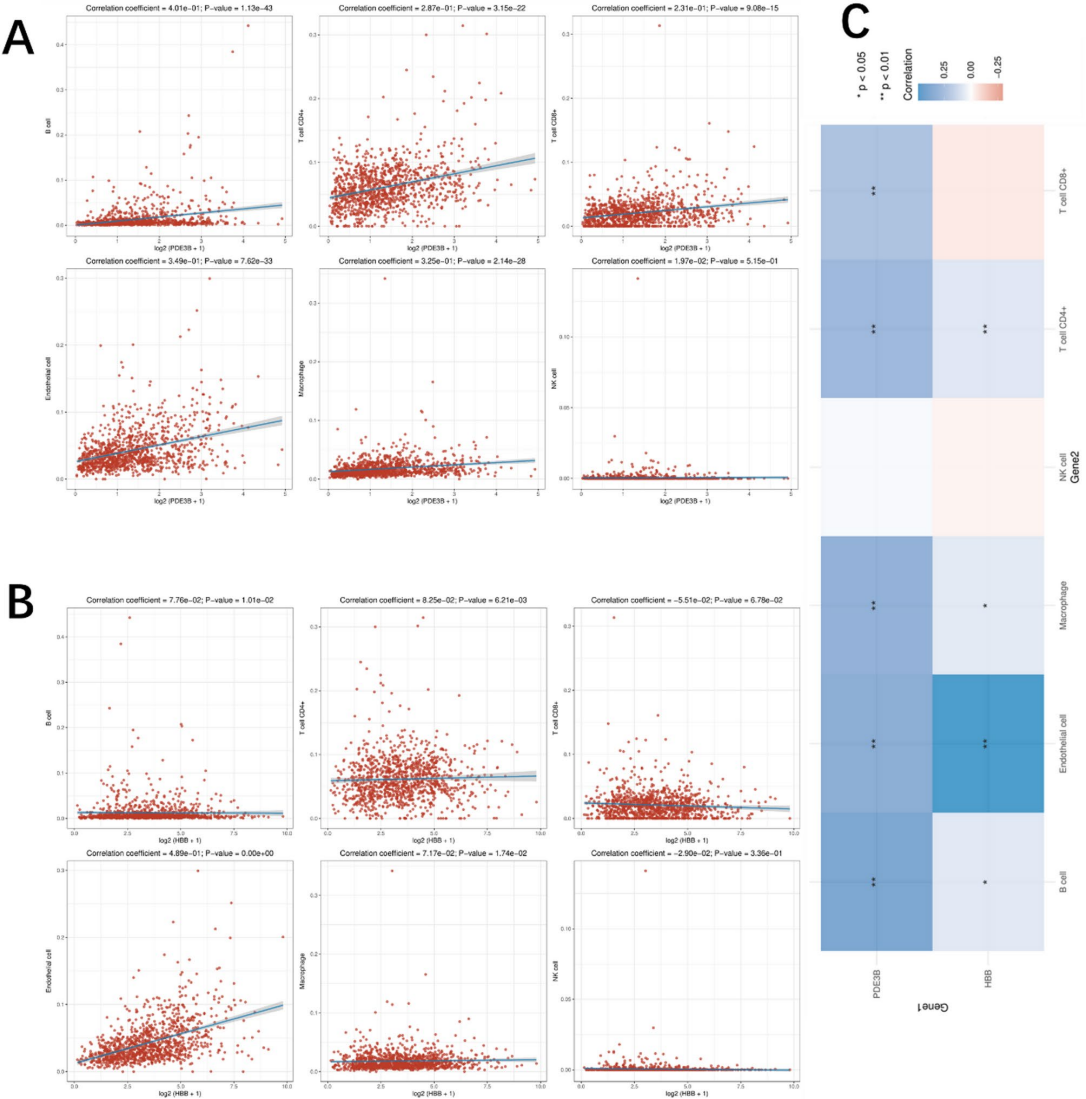
Correlation of PDE3B and HBB expression with immune cell infiltration in TCGA breast cancer samples. (**A**) Scatter plots showing the correlation between PDE3B expression (log2) and various immune cell fractions estimated using EPIC. Correlation coefficients and p-values are indicated in each panel. (**B**) Scatter plots showing the correlation between HBB expression (log2) and immune cell fractions. Correlation coefficients and p-values are indicated in each panel. (**C**) Heatmap summarizing the correlation coefficients between immune cell fractions and the expression of PDE3B and HBB. Blue indicates positive correlations, while red indicates negative correlations. Significant correlations (*p* < 0.05) are marked with asterisks

To investigate the relationship between PDE3B and HBB expression and immune infiltration, we utilized EPIC deconvolution to quantify immune cell fractions in TCGA breast cancer samples. Correlation analysis revealed significant positive associations between PDE3B expression and certain immune cell types, including macrophages and CD4 + T cells (Fig. 4A). Similarly, HBB expression demonstrated significant positive correlations with macrophages and endothelial cells, suggesting potential involvement in immune modulation (Fig. 4B). The heatmap summarizing the correlation coefficients and statistical significance for both genes further highlights the distinct immune cell types associated with PDE3B and HBB expression (Fig. 4C). Although the effect sizes are relatively small, these results indicate that PDE3B and HBB expression may be linked, albeit modestly, to variations in the tumor immune microenvironment, warranting further investigation.

### Internal validation of prognostic significance for PDE3B and HBB in an independent patient cohort

**Fig. 5:**
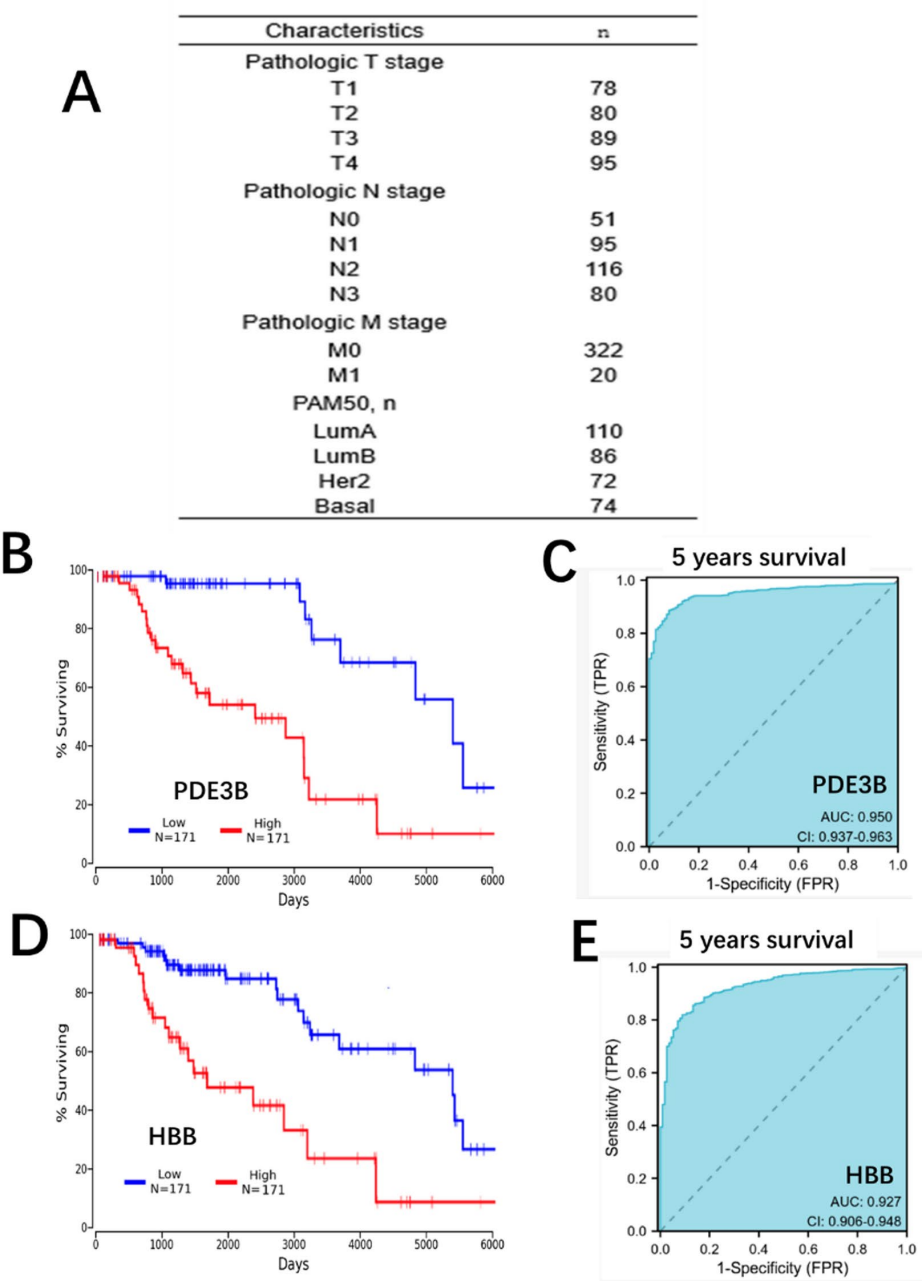
Internal validation of prognostic significance for PDE3B and HBB in an independent patient cohort. (**A**) Clinicopathological characteristics of the independent patient cohort, including tumor stage, lymph node involvement, metastasis stage, and molecular subtypes. (**B**) Kaplan-Meier survival analysis of PDE3B. Patients were stratified into high- and low-expression groups, and overall survival was compared using the log-rank test. (**C**) Kaplan-Meier survival analysis of HBB. Patients were stratified into high- and low-expression groups, and overall survival was compared using the log-rank test. (**D**) Time-dependent ROC curve for 5-year survival prediction based on PDE3B expression. The AUC and confidence interval (CI) are shown. (**E**) Time-dependent ROC curve for 5-year survival prediction based on HBB expression. The AUC and confidence interval (CI) are shown

To validate the prognostic relevance of PDE3B and HBB, we analyzed an independent patient cohort with detailed clinicopathological characteristics (Fig. 5A). The Gene expression level was determined using PCR. Kaplan-Meier survival analysis revealed that higher expression levels of PDE3B and HBB were significantly associated with worse overall survival (*p* < 0.05) (Fig. 5B, C), consistent with findings in previous analyses. Furthermore, the prognostic accuracy of these genes was assessed using time-dependent ROC curves for 5-year survival. The area under the curve (AUC) was 0.950 (95% CI: 0.937–0.963) for PDE3B and 0.927 (95% CI: 0.906–0.948) for HBB, demonstrating excellent predictive performance (Fig. 5D, E). These results validate the robustness and clinical utility of PDE3B and HBB as prognostic biomarkers in breast cancer.

### Immunohistochemistry validation of PDE3B and HBB protein expression

**Fig. 6:**
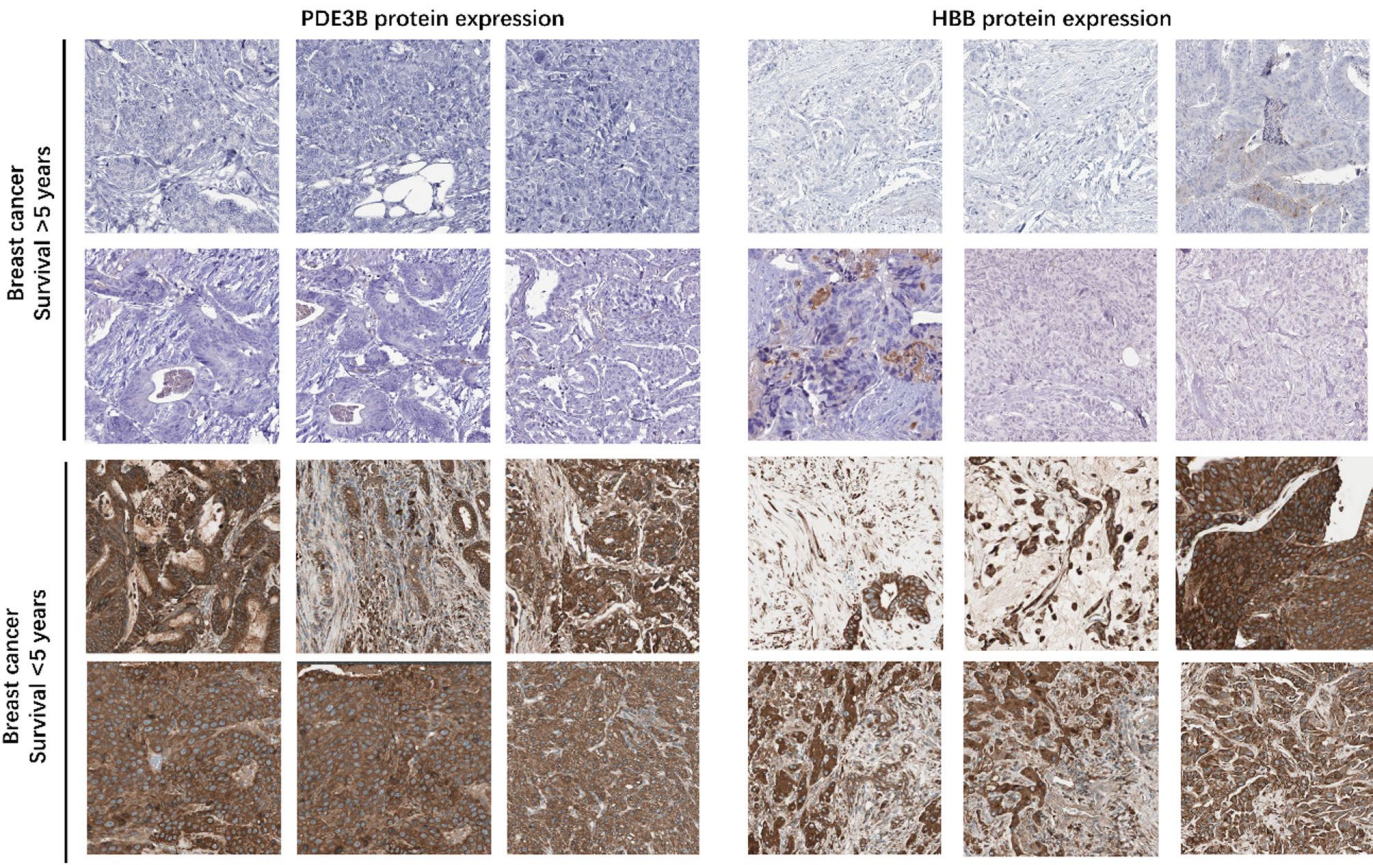
Immunohistochemistry (IHC) analysis of PDE3B and HBB protein expression in breast cancer tissues. Left panel: Representative IHC images of PDE3B expression in low-expression (top) and high-expression (bottom) groups. Stronger staining intensity is observed in the high-expression group. Right panel: Representative IHC images of HBB expression in low-expression (top) and high-expression (bottom) groups. High-expression tissues show stronger staining. Each image represents a 40× magnification of the tissue sections

To validate the protein expression levels of PDE3B and HBB, we performed immunohistochemistry (IHC) on tissue samples from an independent patient cohort. Representative IHC staining images are shown in Fig. 6, with protein expression evaluated in both low-expression and high-expression groups. For PDE3B, tissues from the high-expression group displayed stronger and more diffuse cytoplasmic staining compared to tissues from the low-expression group, indicating elevated protein expression (Fig. 6, left panel). Similarly, HBB showed stronger cytoplasmic staining in the high-expression group compared to the low-expression group, consistent with the gene expression data (Fig. 6, right panel). These findings confirm that both PDE3B and HBB are differentially expressed at the protein level, supporting their potential as prognostic biomarkers in breast cancer.

### Validation of PDE3B expression in cell lines and its association with cell proliferation

**Fig. 7:**
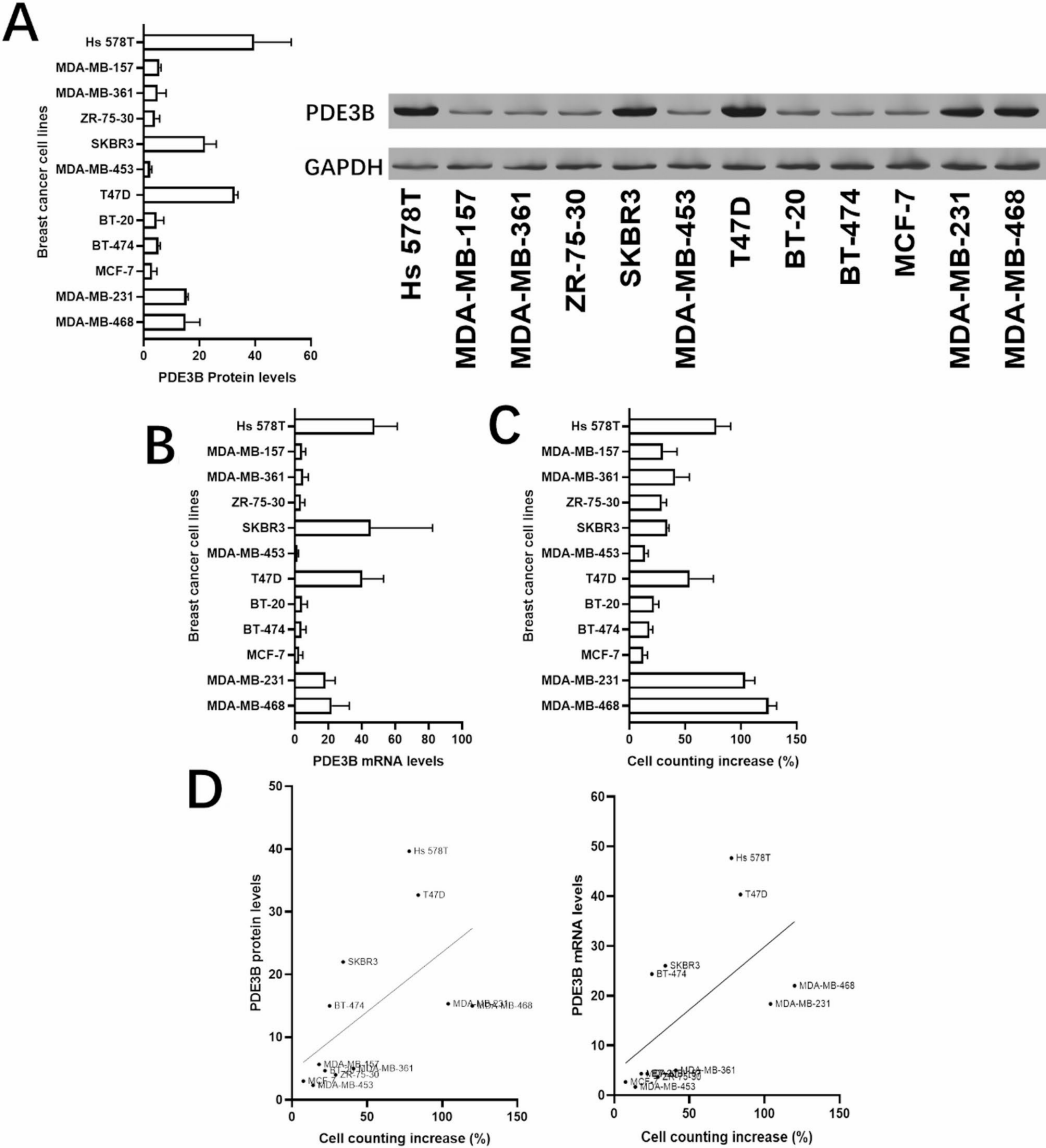
Validation of PDE3B protein expression in breast cancer cell lines and its association with cell proliferation. (**A**) Western blot analysis of PDE3B protein expression in breast cancer cell lines. GAPDH served as the loading control. (**B**) Quantification of PDE3B protein expression levels from the Western blot results. (**C**) Scatter plot showing the positive correlation between PDE3B expression levels and cell proliferation rates in breast cancer cell lines

To explore the role of PDE3B in breast cancer cell proliferation, we performed Western blot analysis across multiple breast cancer cell lines. The results demonstrated variable expression levels of PDE3B among the cell lines, with higher expression observed in cell lines with higher proliferation rates (Fig. 7A). GAPDH was used as a loading control to confirm equal protein loading. Quantification of the Western blot data revealed a clear trend of increased PDE3B expression in highly proliferative cell lines (Fig. 7B). Furthermore, a scatter plot analysis confirmed a positive correlation between PDE3B protein levels and cell proliferation rates (Fig. 7C), suggesting that PDE3B expression may promote cellular proliferation in breast cancer cells. These findings provide additional evidence for the role of PDE3B in supporting tumor progression by enhancing cell proliferation.

### Validation of HBB expression in breast cancer cell lines and its association with cell proliferation

**Fig. 8:**
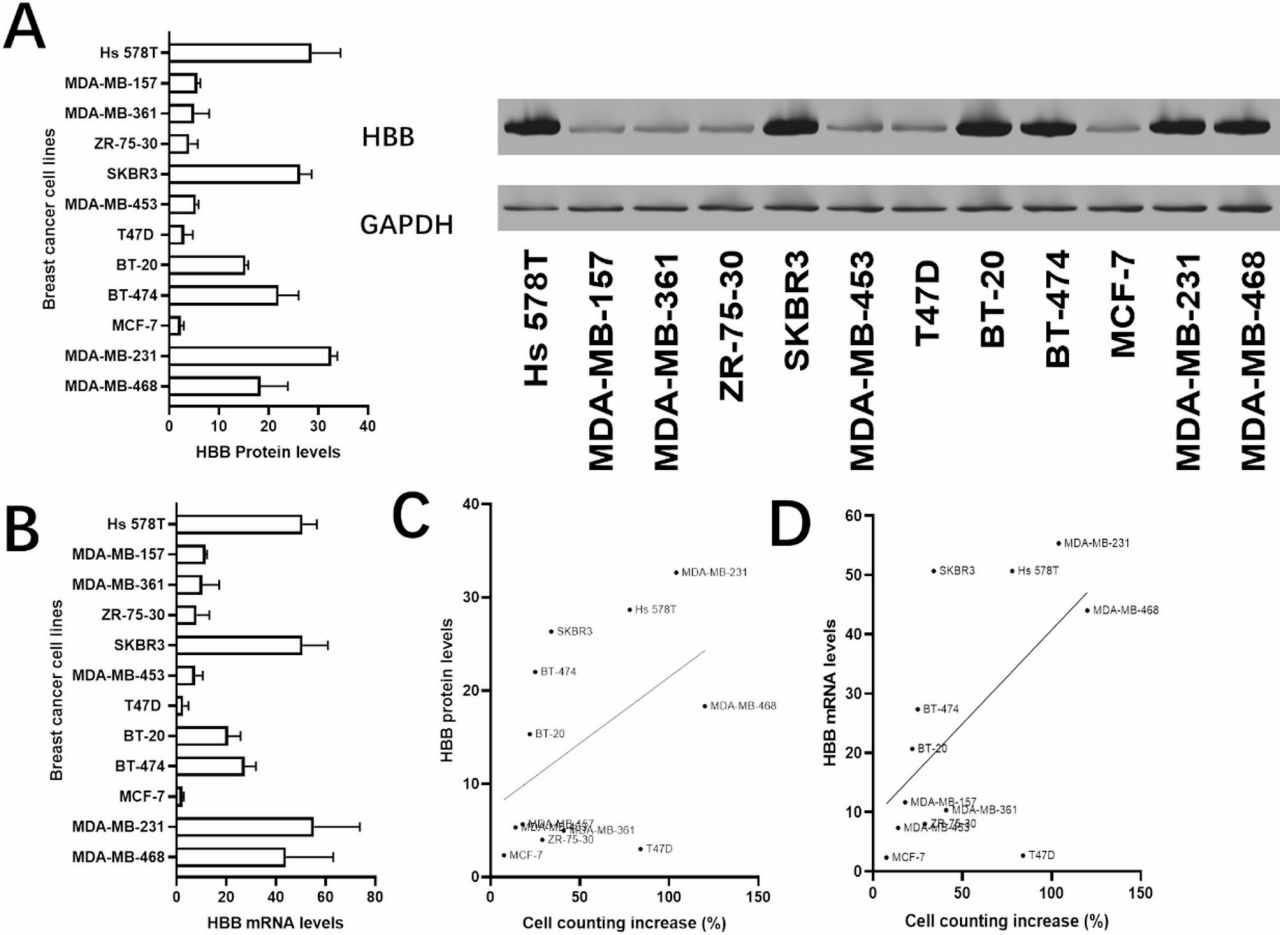
Validation of HBB protein expression in breast cancer cell lines and its association with proliferation. (**A**) Western blot analysis of HBB protein expression in breast cancer cell lines. GAPDH was used as the loading control. (**B**) Quantification of HBB protein expression levels from the Western blot results. (**C**) Scatter plot showing the positive correlation between HBB expression levels and cell proliferation rates in breast cancer cell lines

To further evaluate the role of HBB in breast cancer, we analyzed its protein expression across multiple breast cancer cell lines using Western blotting. The results showed varying levels of HBB expression, with higher expression observed in cell lines exhibiting greater proliferation potential (Fig. 8A). GAPDH was used as a loading control to ensure equal protein loading. Quantitative analysis of the Western blot data revealed that HBB expression positively correlates with cell proliferation rates, as evidenced by increased expression in highly proliferative cell lines (Fig. 8B). Additionally, a scatter plot analysis confirmed this positive correlation between HBB protein levels and proliferation rates (Fig. 8C). These results suggest that HBB may promote tumor growth by enhancing cellular proliferation in breast cancer.

### Validation of PDE3B and HBB roles in proliferation in MDA-MB-231 cells using overexpression and knockout models

**Fig. 9:**
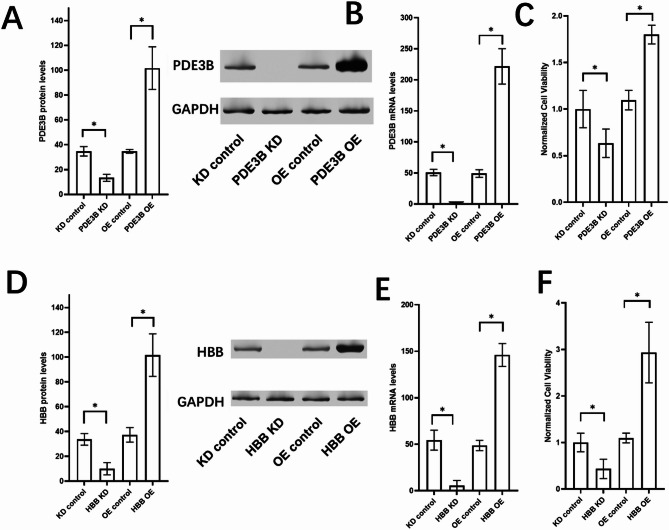
Functional validation of PDE3B and HBB in cell proliferation in MDA-MB-231 cells. (**A**) Western blot analysis confirming the overexpression (OE) and knockout (KO) of PDE3B in MDA-MB-231 cells. GAPDH was used as the loading control. (**B**) Western blot analysis confirming the overexpression (OE) and knockout (KO) of HBB in MDA-MB-231 cells. GAPDH was used as the loading control. (**C**) CCK-8 assay results showing the effect of PDE3B overexpression and knockout on cell proliferation in MDA-MB-231 cells. Data are presented as mean ± standard deviation. (**D**) CCK-8 assay results showing the effect of HBB overexpression and knockout on cell proliferation in MDA-MB-231 cells. Data are presented as mean ± standard deviation

To explore the roles of PDE3B and HBB in breast cancer proliferation, we established overexpression (OE) and knockout (KO) models in MDA-MB-231 cells. Western blot analysis confirmed the successful overexpression and knockout of PDE3B (Fig. 9A) and HBB (Fig. 9B), with GAPDH as the loading control. We assessed cell proliferation using the CCK-8 assay. Overexpression of PDE3B significantly increased proliferation in MDA-MB-231 cells, whereas knockout of PDE3B led to a marked reduction in proliferation (Fig. 9C). Similarly, HBB overexpression enhanced proliferation, while knockout of HBB suppressed cell growth (Fig. 9D). These results demonstrate that both PDE3B and HBB play critical roles in promoting the proliferation of MDA-MB-231 cells.

## Discussion

In this study, we identified and validated PDE3B and HBB as potential prognostic biomarkers in breast cancer through integrative bioinformatic analyses and experimental validation. By combining single-cell RNA-seq data, TCGA datasets, and an independent patient cohort, we explored malignant cell heterogeneity, proliferative potential, and interactions with the immune microenvironment. Our findings shed light on the molecular mechanisms driving tumor progression and highlight PDE3B and HBB as promising therapeutic targets.

Breast cancer is a heterogeneous disease with complex interactions between tumor cells and their microenvironment [8, 9]. Our study integrates single-cell RNA-seq, TCGA data analysis, and experimental validation to investigate the roles of PDE3B and HBB in breast cancer proliferation and their potential as prognostic biomarkers. The findings not only corroborate previous research on tumor heterogeneity but also provide novel insights into the functional roles of PDE3B and HBB in driving breast cancer progression.

Single-cell RNA-seq analysis identified significant heterogeneity among malignant cell subpopulations, consistent with prior studies demonstrating the diverse phenotypes of tumor cells in breast cancer [10, 11]. For instance, a study emphasized that distinct malignant cell subpopulations are associated with specific functional states, including proliferation, immune evasion, and therapy resistance [12]. Our findings expand on this by showing that malignant cell type 3 exhibited the highest proliferative capacity among the identified subpopulations. KEGG pathway enrichment analysis of this cell type revealed significant enrichment in the cell cycle and DNA replication pathways, further validating its aggressive phenotype. These results suggest that certain malignant cell subpopulations may serve as critical drivers of tumor growth and could be targeted therapeutically.

Through integrative analysis of TCGA data and single-cell RNA-seq, PDE3B and HBB were identified as key prognostic genes. Elevated expression of these genes was significantly associated with poor overall survival in both TCGA and our independent patient cohort. Prior studies have linked PDE3B to metabolic regulation and its dysregulation in cancers such as pancreatic and ovarian cancer (Smith et al., 2019). However, its role in breast cancer remained largely unexplored. Our findings provide the first evidence that PDE3B not only correlates with breast cancer proliferation but also serves as a robust prognostic marker. Similarly, HBB, primarily known for its role in hemoglobin synthesis, has been implicated in tumor growth and metastasis in other cancer types. A recent study highlighted the pro-tumorigenic role of HBB in lung cancer [7], supporting our results that HBB promotes proliferation in breast cancer cells.

Both PDE3B and HBB were significantly associated with immune cell infiltration in the tumor microenvironment. Specifically, PDE3B expression correlated positively with macrophages and CD4 + T cells, while HBB was associated with macrophages and endothelial cells. Immune cell infiltration has been shown to impact tumor progression and patient outcomes. For instance, macrophage infiltration has been associated with tumor-promoting inflammation and immune suppression [13]. Our results suggest that PDE3B and HBB may modulate the immune microenvironment, potentially contributing to tumor immune evasion and progression. This hypothesis warrants further investigation, as targeting these genes could simultaneously disrupt tumor growth and reprogram the immune microenvironment.

Experimental validation using MDA-MB-231 cells demonstrated that both PDE3B and HBB significantly promote breast cancer cell proliferation. Knockout of these genes markedly reduced proliferation, while overexpression enhanced it, as confirmed by Western blot and CCK-8 assays. These findings align with prior reports linking these genes to cancer progression but provide a more detailed functional perspective in the context of breast cancer. For example, Begum N et al. (2022) demonstrated that PDE3B regulates cell cycle progression [14], and our study extends this role to breast cancer, particularly in highly proliferative subpopulations. Similarly, our findings on HBB build upon its previously reported pro-tumorigenic effects in other cancers by showing its direct involvement in breast cancer proliferation.

The strong association of PDE3B and HBB with tumor cell proliferation and patient survival underscores their utility as prognostic biomarkers and therapeutic targets. Time-dependent ROC curve analysis demonstrated excellent predictive accuracy for 5-year overall survival, with AUCs of 0.950 for PDE3B and 0.927 for HBB—highlighting their clinical relevance. Moreover, both genes appear to modulate the tumor immune microenvironment, suggesting that combining PDE3B or HBB inhibition with immune checkpoint blockade may yield synergistic antitumor effects. Future preclinical and clinical studies should investigate these combination strategies to fully realize their therapeutic potential.

The Cancer Genome Atlas (TCGA) has been an invaluable resource for cancer biomarker discovery, enabling large-scale investigations of genetic, transcriptomic, and epigenomic alterations across multiple cancer types. While TCGA data offer unparalleled insights into cancer biology, several biases must be considered [15, 16]. Bulk transcriptomic data can obscure cellular heterogeneity and immune infiltration, limiting their ability to capture the full complexity of tumor microenvironments. Our use of scRNA-seq complements TCGA by resolving this heterogeneity, particularly in malignant cell subpopulations. However, our study is not without limitations. TCGA datasets predominantly include primary tumors, with limited representation of metastatic or treatment-resistant samples. Additionally, the reliance on bulk RNA-seq data for differential expression analysis introduces potential confounding factors, such as stromal contamination and sample heterogeneity [17, 18]. Future studies should address these biases by incorporating spatial transcriptomics or single-cell multi-omics approaches to provide a more nuanced understanding of tumor biology.

In future studies, it will be important to develop and employ bespoke in vitro models that more faithfully recapitulate the biology of breast cancer subtypes. For example, specialized primary cell lines or organoid systems derived directly from patient tumors can preserve key histological and molecular features, enabling more accurate functional assays of gene candidates such as PDE3B and HBB. A notable precedent is the work by Zhang et al., who established breast phyllodes tumor cell lines that retain the characteristic stromal–epithelial interactions and gene expression profiles of the original tumors [19]. Adapting a similar strategy to generate subtype-specific breast cancer models—be they 2D cell lines, 3D spheroids, or patient-derived organoids—would provide a powerful platform to dissect the mechanistic roles of PDE3B and HBB in tumor growth, proliferation, and immune crosstalk.

## Conclusion

In summary, our study identifies PDE3B and HBB as key drivers of breast cancer cell proliferation and predictors of poor prognosis. By integrating single-cell RNA-seq and TCGA data with experimental validation, we provide novel insights into their roles in tumor heterogeneity and modulation of the immune microenvironment. These findings not only enhance our understanding of breast cancer biology but also highlight PDE3B and HBB as promising prognostic biomarkers and therapeutic targets, paving the way for future research and clinical applications.

## Electronic supplementary material

Below is the link to the electronic supplementary material.

Supplementary Material 1

Supplementary Material 2

## Data Availability

No datasets were generated or analysed during the current study.
