# Supplementary figures and images for: PDE3B and HBB are key prognostic biomarkers driving cell proliferation and regulating immune microenvironment in breast cancer
Source: Hereditas. 2025 Jun 3;162:97. doi: 10.1186/s41065-025-00470-z (PMC12131617; doi:10.1186/s41065-025-00470-z)

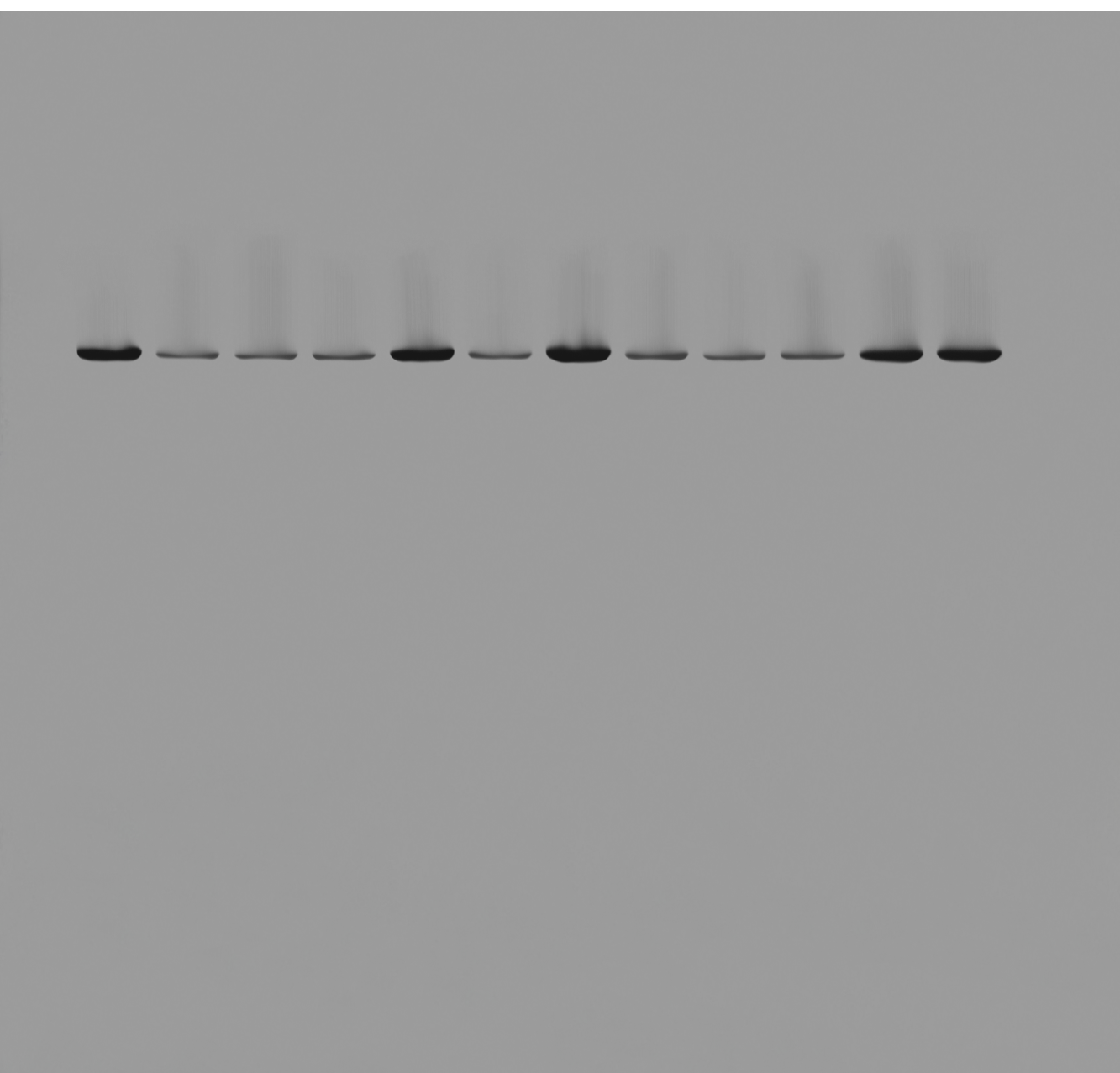

Supplement: Supplementary file 1 — Supplementary Material 1 [file 41065_2025_470_MOESM1_ESM.zip › WB/2.1.png]

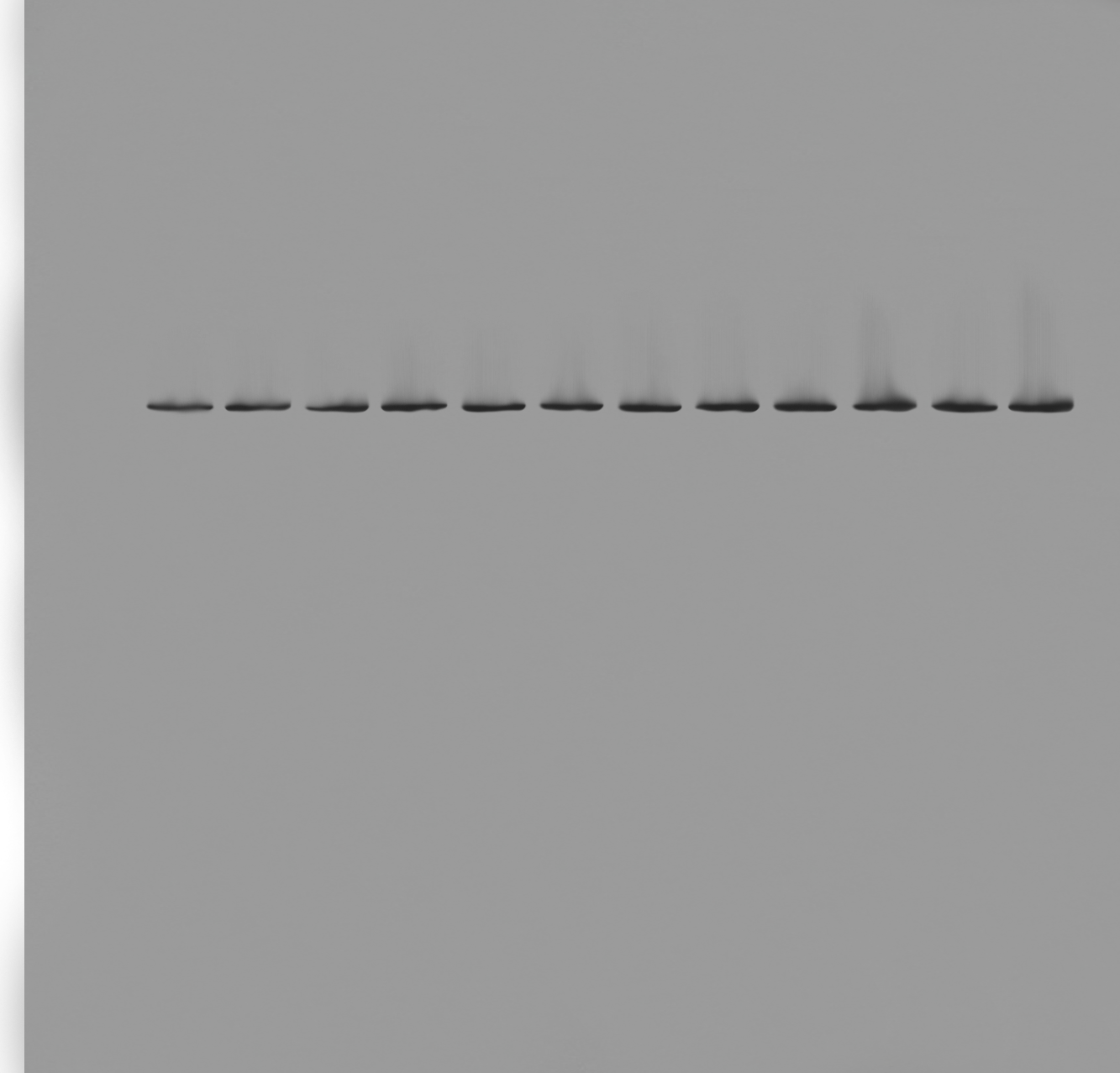

Supplement: Supplementary file 1 — Supplementary Material 1 [file 41065_2025_470_MOESM1_ESM.zip › WB/2.2.png]

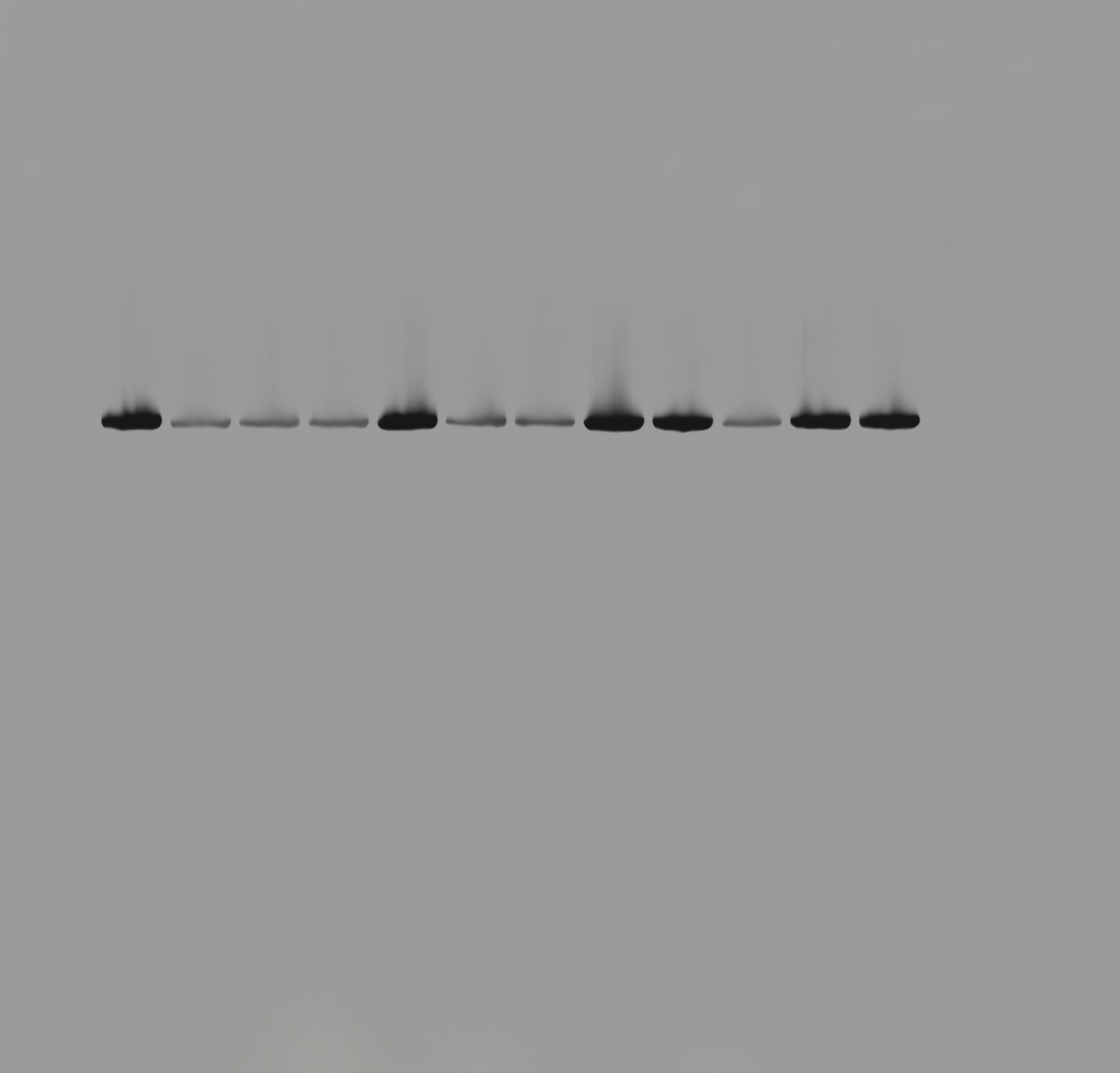

Supplement: Supplementary file 1 — Supplementary Material 1 [file 41065_2025_470_MOESM1_ESM.zip › WB/3.1.png]

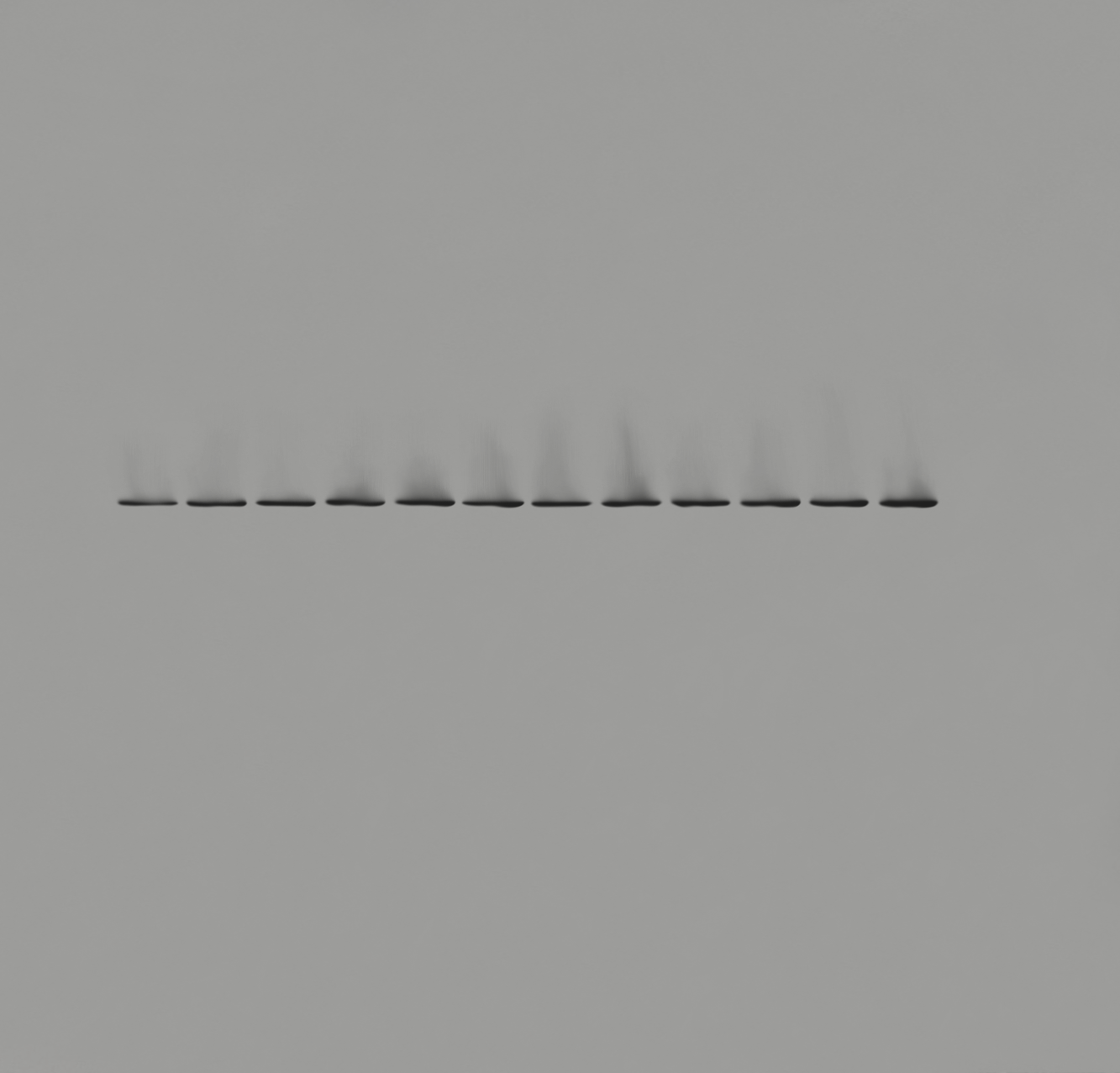

Supplement: Supplementary file 1 — Supplementary Material 1 [file 41065_2025_470_MOESM1_ESM.zip › WB/3.2.png]

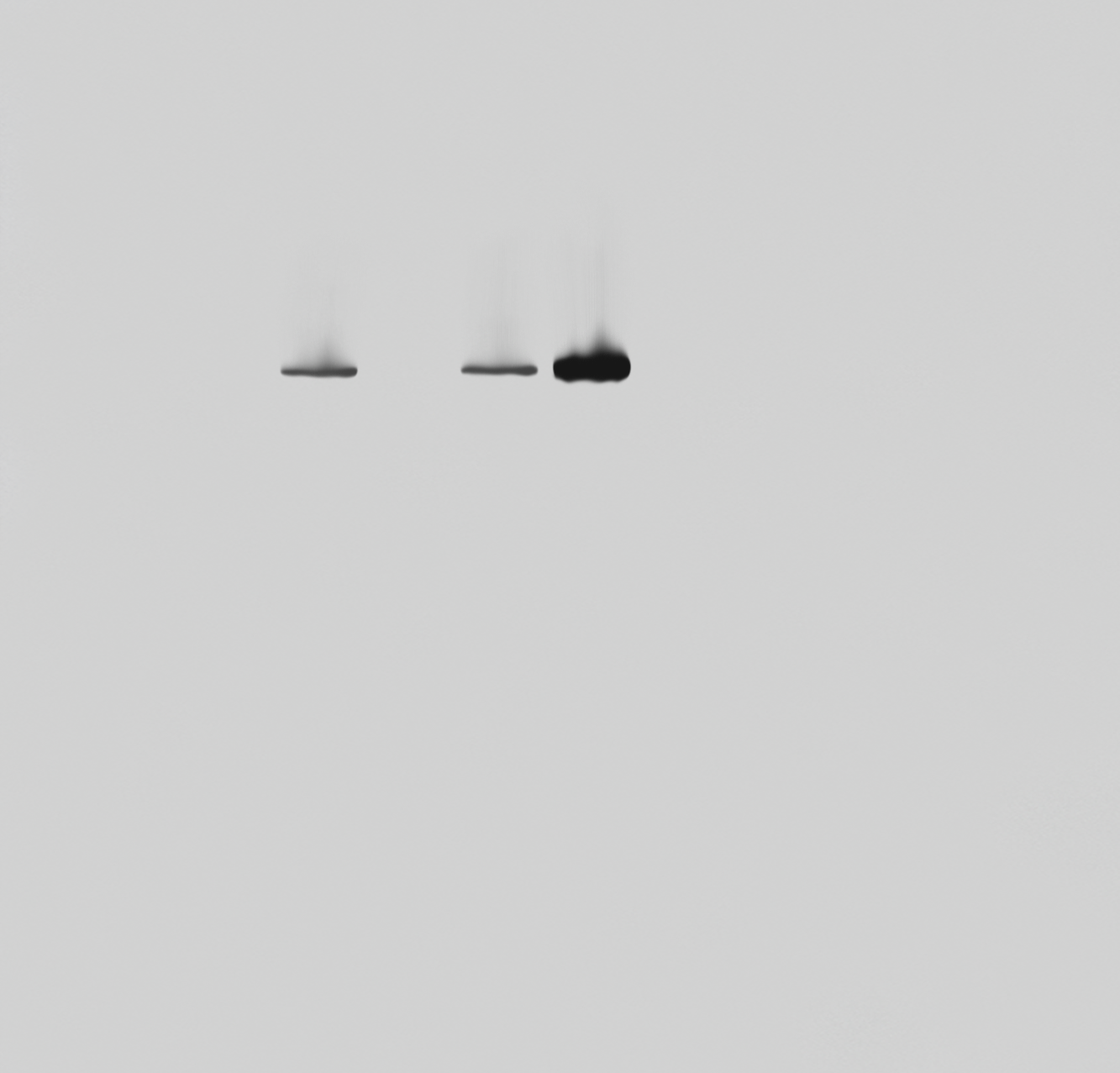

Supplement: Supplementary file 1 — Supplementary Material 1 [file 41065_2025_470_MOESM1_ESM.zip › WB/4.1.png]

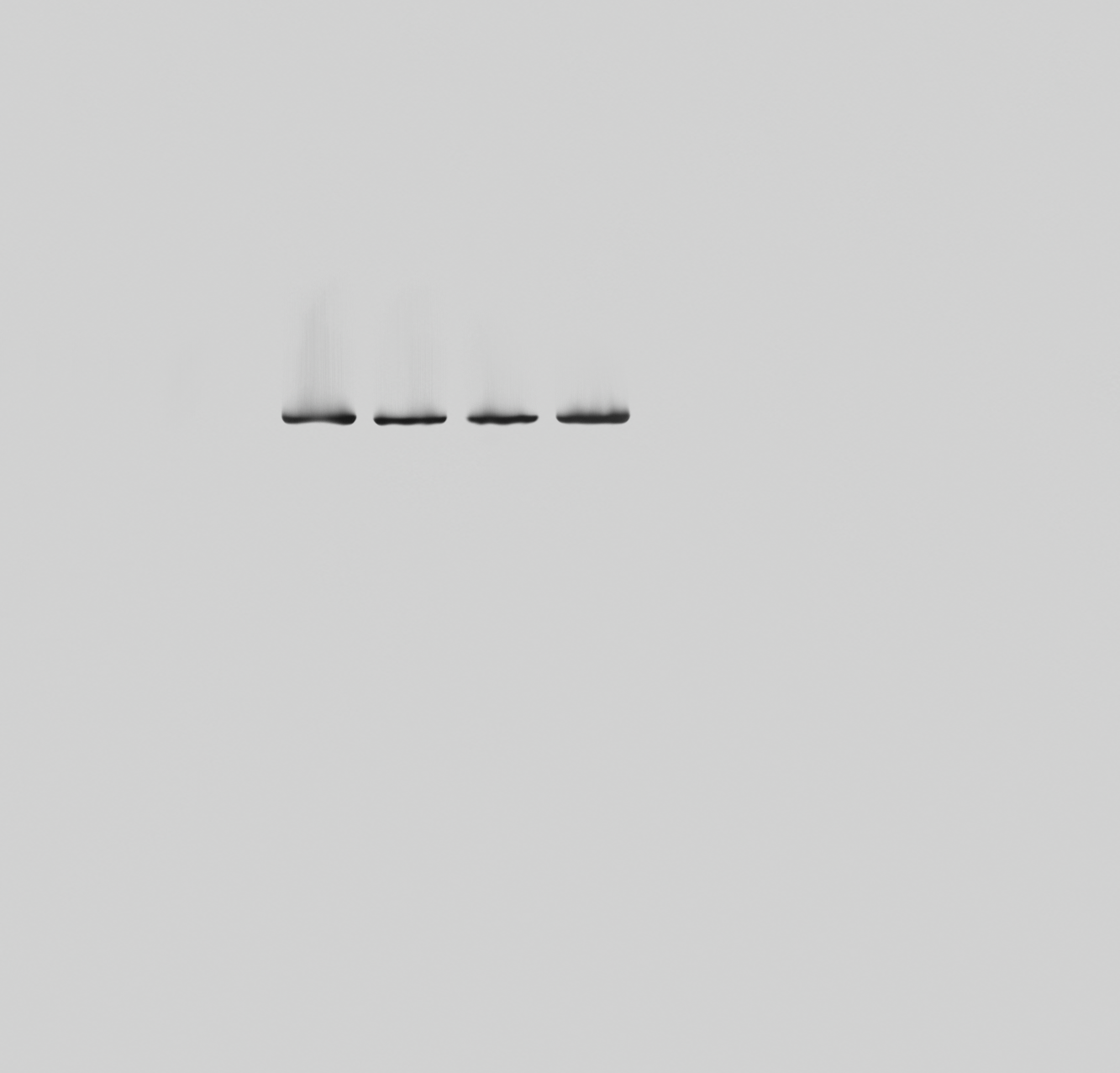

Supplement: Supplementary file 1 — Supplementary Material 1 [file 41065_2025_470_MOESM1_ESM.zip › WB/4.2.png]

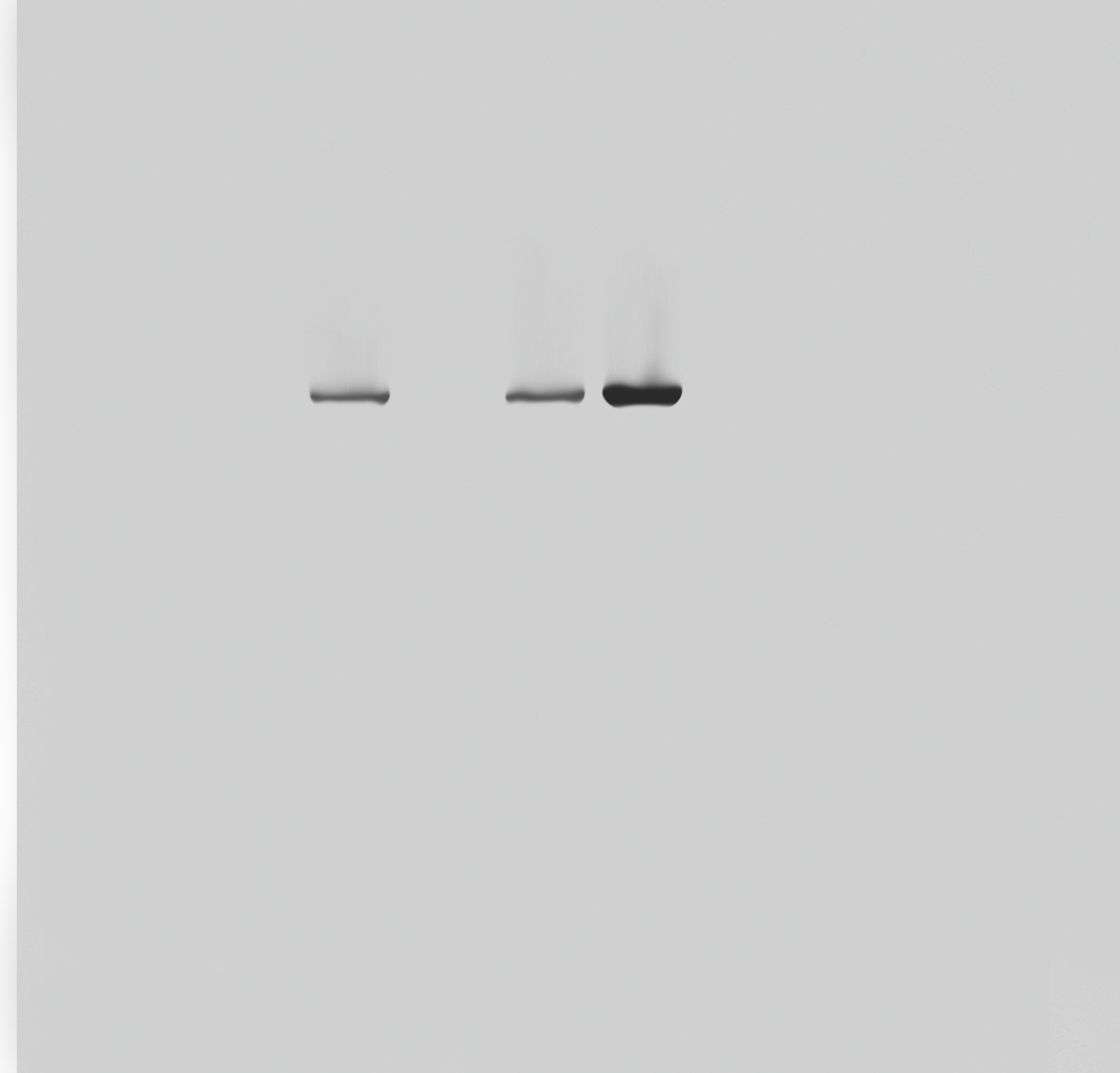

Supplement: Supplementary file 1 — Supplementary Material 1 [file 41065_2025_470_MOESM1_ESM.zip › WB/4.3.png]

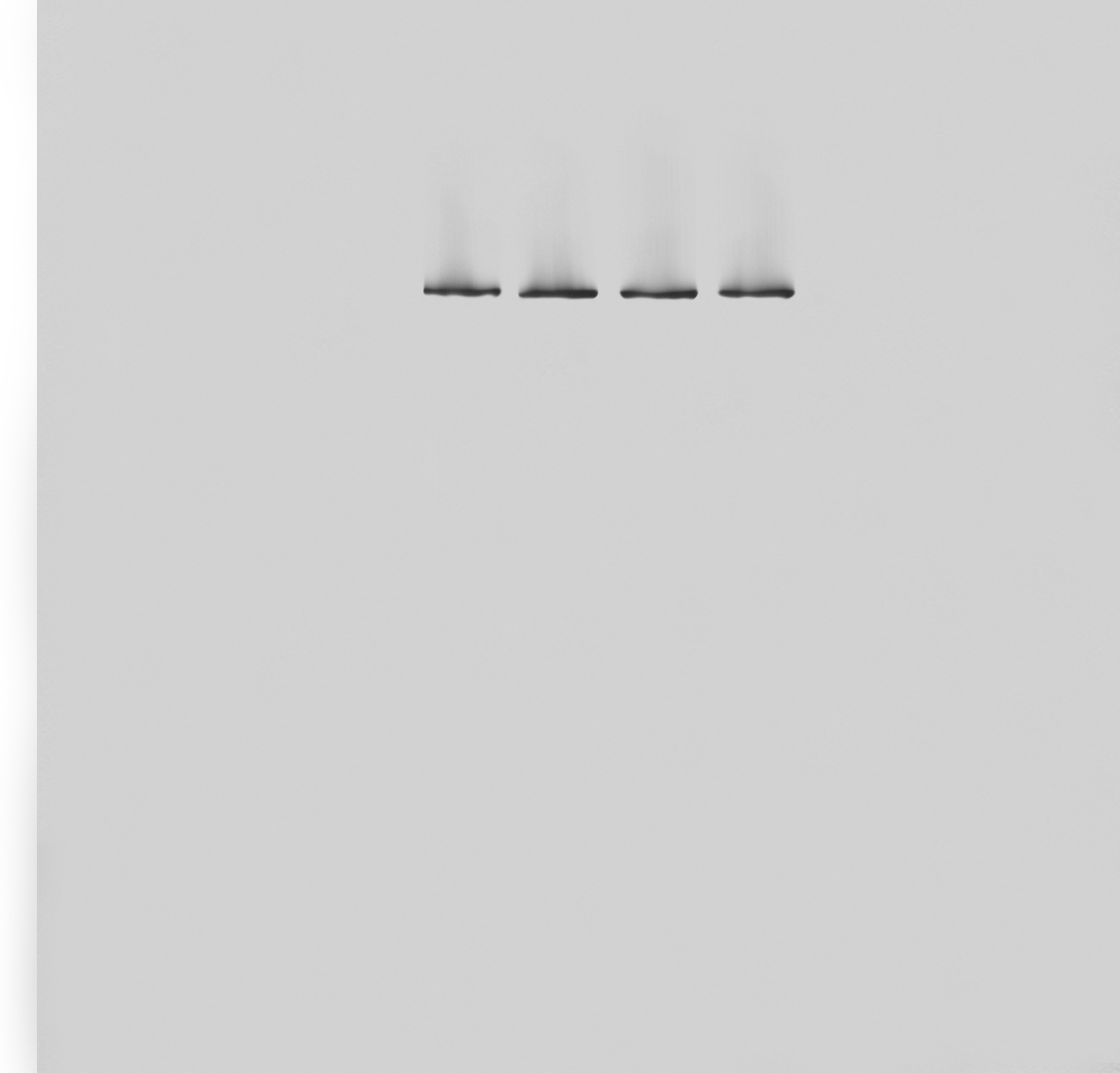

Supplement: Supplementary file 1 — Supplementary Material 1 [file 41065_2025_470_MOESM1_ESM.zip › WB/4.4.png]

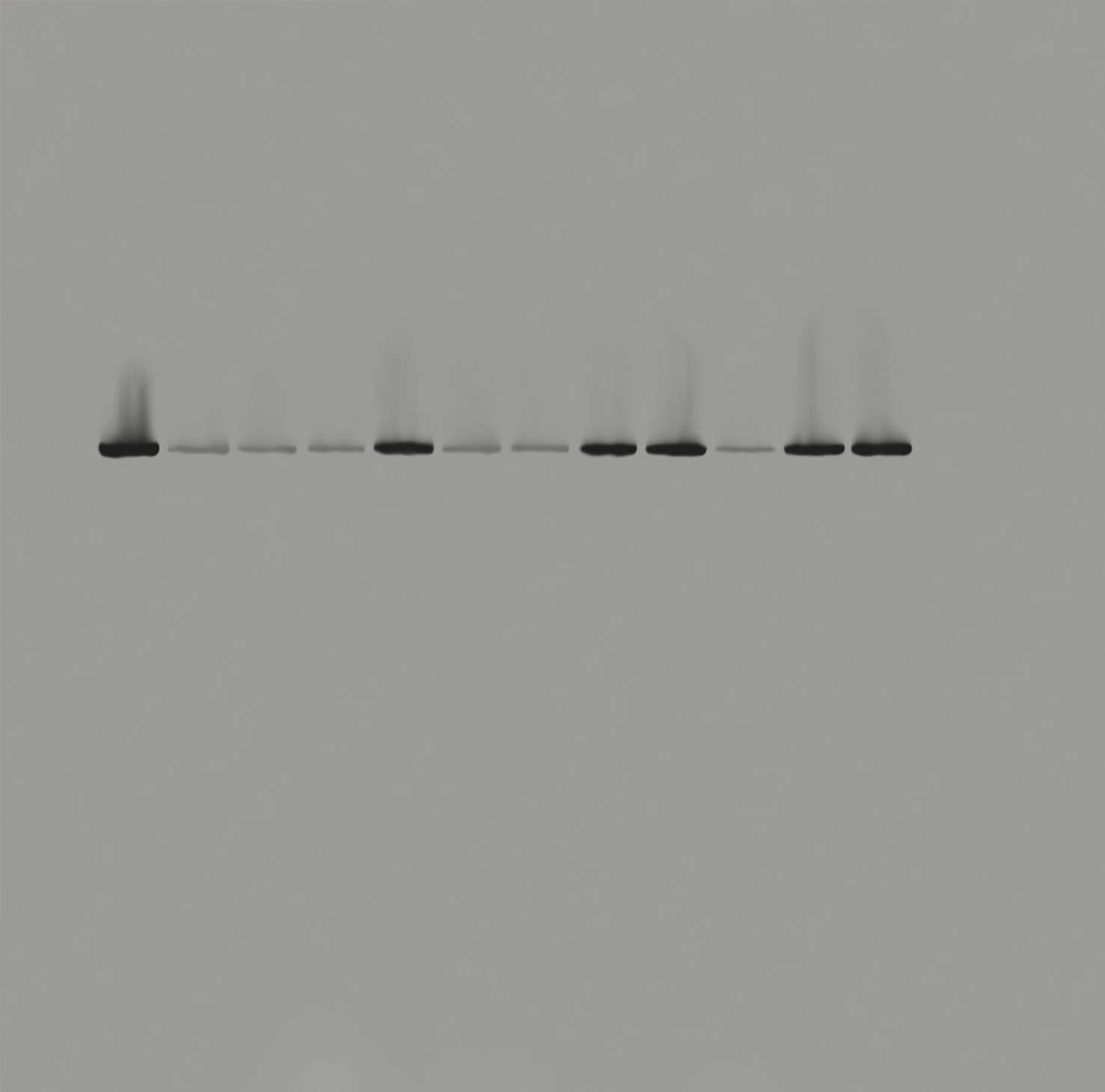

Supplement: Supplementary file 1 — Supplementary Material 1 [file 41065_2025_470_MOESM1_ESM.zip › WB/b.png]
