# Supplementary material for: PDE3B and HBB are key prognostic biomarkers driving cell proliferation and regulating immune microenvironment in breast cancer
Source: Hereditas. 2025 Jun 3;162:97. doi: 10.1186/s41065-025-00470-z (PMC12131617; doi:10.1186/s41065-025-00470-z)
Supplement: Supplementary file 2 — Supplementary Material 2 [file 41065_2025_470_MOESM2_ESM.docx]

| **Gene Symbol** | **Gene Name** | **Functional Annotation** |
| --- | --- | --- |
| MKI67 | Marker of Proliferation Ki-67 | Cellular proliferation marker |
| PCNA | Proliferating Cell Nuclear Antigen | DNA replication accessory factor |
| TOP2A | DNA Topoisomerase II Alpha | Relieves DNA supercoiling during replication |
| CCNB1 | Cyclin B1 | Regulates G₂/M transition |
| CCNB2 | Cyclin B2 | Regulates G₂/M transition |
| CCNA2 | Cyclin A2 | Controls S-phase and G₂-phase progression |
| CDK1 | Cyclin-Dependent Kinase 1 | Drives entry into mitosis |
| CDC20 | Cell Division Cycle 20 | Activates APC/C complex for mitotic progression |
| CDC25A | Cell Division Cycle 25A | Dephosphorylates CDK1 to promote G₂/M entry |
| MCM2 | Minichromosome Maintenance Complex Component 2 | Part of the DNA helicase complex |
| MCM3 | Minichromosome Maintenance Complex Component 3 | Part of the DNA helicase complex |
| MCM4 | Minichromosome Maintenance Complex Component 4 | Part of the DNA helicase complex |
| MCM5 | Minichromosome Maintenance Complex Component 5 | Part of the DNA helicase complex |
| MCM6 | Minichromosome Maintenance Complex Component 6 | Part of the DNA helicase complex |
| MCM7 | Minichromosome Maintenance Complex Component 7 | Part of the DNA helicase complex |
| RRM2 | Ribonucleotide Reductase Regulatory Subunit M2 | Key enzyme for dNTP synthesis |
| TYMS | Thymidylate Synthetase | Essential for DNA synthesis |
| PTTG1 | Pituitary Tumor-Transforming 1 | Involved in securin regulation and spindle checkpoint |
| AURKA | Aurora Kinase A | Spindle assembly and chromosome segregation |
| AURKB | Aurora Kinase B | Regulates metaphase–anaphase transition and |
